# Prp19 Is an Independent Prognostic Marker and Promotes Neuroblastoma Metastasis by Regulating the Hippo-YAP Signaling Pathway

**DOI:** 10.3389/fonc.2020.575366

**Published:** 2020-11-02

**Authors:** Yuanxia Cai, Kai Chen, Cheng Cheng, Yonghu Xu, Qianqian Cheng, Guofeng Xu, Yeming Wu, Zhixiang Wu

**Affiliations:** ^1^Department of Pediatric Surgery, Xinhua Hospital, School of Medicine, Shanghai Jiaotong University, Shanghai, China; ^2^Division of Pediatric Oncology, Shanghai Institute of Pediatric Research, Shanghai, China; ^3^Department of Pediatric Urology, Xinhua Hospital, National Key Clinical Specialty, Shanghai Top-Priority Clinical Center, School of Medicine, Shanghai Jiaotong University, Shanghai, China; ^4^Department of Pediatric Surgery, Children's Hospital of Soochow University, Suzhou, China

**Keywords:** Prp19, neuroblastoma, metastasis, YAP, RNA splicing

## Abstract

Pre-mRNA processing factor 19 (Prp19) was previously reported to be involved in tumor progression. However, Prp19 expression and its functions remain elusive in neuroblastoma. Here, we aim to identify the functions and mechanisms of Prp19 in neuroblastoma. Neuroblastic tumor tissue microarrays and two independent validation data sets indicate that Prp19 is associated with high-risk markers and bone marrow metastasis and serves as a prognostic marker for worse clinical outcomes with neuroblastoma. Gain- and loss-of-expression assays reveal that Prp19 promotes invasion, migration, and epithelial–mesenchymal transition (EMT) of neuroblastoma cells *in vitro*. Bioinformatics analysis of RNA-seq data shows that the expressions of YAP and its downstream genes are significantly inhibited after downregulation of Prp19. Prp19 and YAP expression in metastatic lymph nodes is higher than *in situ* neuroblastoma tissue. Further experiments show that Prp19 regulates YAP expression and consequently affects cell invasion, migration, and EMT in neuroblastoma by pre-mRNA splicing of YAP. In conclusion, our findings provide the first evidence that Prp19 is a potential therapeutic target and prognostic biomarker for patients with neuroblastoma.

## Introduction

Neuroblastoma, arising from neural crest progenitor cells of the sympathoadrenal lineage, is the most common extracranial solid tumor in children, accounting for 7.5% of all childhood cancers and 11–15% of all childhood cancer-related deaths (1, 2). The most prominent characteristic of neuroblastoma is extreme heterogeneity, which ranges from spontaneous regression in infants to metastasis and progression in older children despite intensive multimodality therapy. Children with limited lesions usually have a good prognosis, and the 5-year event-free survival (EFS) rate can reach 83% (3). However, the long-term prognosis for patients with distant metastasis is poor with a 5-year EFS of 35%, which has not improved in the last decades (3–5). A better understanding of the biological mechanism of metastatic neuroblastoma will likely refine treatment strategies and further improve the prognosis of metastatic patients.

Pre-mRNA processing factor 19 (Prp19)-associated complex (Prp19C) is a highly conserved multiprotein complex (6). As an important member of Prp19C (7), Prp19 has many vital biological functions, such as splicing of pre-mRNA, cell cycle regulation, DNA damage repair, protein degradation, and metastasis (8–13). Because these intracellular events are closely related to cell fate, aberrant Prp19 function may cause serious diseases, for example, cancer. In fact, studies have found Prp19 expression is higher in colon, larynx, and hepatocellular carcinoma compared with paracancerous tissues and is positively correlated with poor prognosis (13, 14). However, the function of Prp19 in tumors and its association with poor prognosis have not been well-demonstrated. Increasing evidence suggests that abnormalities in splicing events may involve tumorigenesis and development. For example, hnRNPK promotes gastric tumorigenesis through regulating CD44E alternative splicing (15). Alternative splicing of EZH2 pre-mRNA by SF3B3 contributes to the tumorigenic potential of renal cancer (16). As an important splicing factor, Prp19 may also participate in tumorigenesis and development by regulating alternative splicing of some key molecules.

The Hippo-YAP pathway is a highly conserved signaling pathway that functions in the regulation of organ size, cell proliferation, invasion, and metastasis (17, 18). When the Hippo-YAP signaling is off, YAP, the key factor in the Hippo pathway, translocates into the nucleus to drive transcription of downstream genes, promoting of cell proliferation, migration, and tumor growth. When Hippo-YAP is on, YAP is phosphorylated and retained in the cytoplasm, turning off downstream target gene expression (17). In addition to the classic Hippo-YAP signaling, YAP expression and stability can be regulated by a variety of molecules, such as CD44, HER3, and Fbxw7 (19–22). The mechanism governing YAP protein expression by alterative splicing remains poorly understood. This study presents a lot of overlap between the functions of Prp19 and YAP. On account of this, whether there is a regulatory effect between Prp19 and YAP is worth further exploration.

Our investigation into the expression and potential role and mechanism of Prp19 in neuroblastoma reveals that the expression of Prp19 is positively correlated with bone marrow metastasis of neuroblastoma and that Prp19 promotes neuroblastoma invasion and migration *in vitro*. Also, YAP is identified as a candidate target of Prp19 because the mRNA maturity of it is regulated by Prp19. In metastatic lymph nodes, Prp19 and YAP show obvious higher expression than their paired primary tumor. Taken together, these results indicate that Prp19 promotes neuroblastoma metastasis via increasing pre-mRNA splicing to upregulate the level of YAP.

## Materials and Methods

### Patients and Tissue Specimens

This study includes 62 pediatric patients with neuroblastoma who were diagnosed and treated in Xinhua Hospital, a subsidiary hospital of Shanghai Jiaotong University School of Medicine, from September 2012 to February 2015. The patient group includes 43 neuroblastoma/gneuroblastoma-N (NB/GNB-N) and 19 gneuroblastoma-I (GNB-I) cases. A total of 4 pairs of neuroblastoma *in situ* and their corresponding metastatic lymph node tissues were collected. The clinical and prognosis information of the patients was collected from recorded clinical data and follow-up through phone calls; data include gender, age at diagnosis, bone marrow metastasis, clinical stage, diagnostic category, Shimada pathologic type, and risk classification. All patients underwent surgery or biopsy in our hospital, and each tumor specimen was stored in liquid nitrogen until tissue microarray (TMA) analysis. The experimental protocols were approved by the Ethics Committee of the Xinhua Hospital affiliated to Shanghai Jiaotong University School of Medicine.

### TMA Preparation and Immunohistochemistry (IHC)

IHC was performed as previously described (23). IHC staining was performed using a standard immunoperoxidase staining procedure, and the primary antibody included Prp19 (1:200, Abcam, ab126776) and YAP (ab52771, 1:50, abcam). Hematoxylin was used as a counterstain. The tissue sections were viewed independently by two pathologists in a double-blind fashion. IHC staining was graded on a specialized scale from 0 to 4, where 0 represents negative expression, 1 represents weakly positive expression (0–10% positive cells), 2 represents mildly positive expression (10–30% positive cells), 3 represents moderately positive expression (30–50% positive cells), and 4 represents strongly positive expression (50–100% positive cells). The scale was determined according to the average number of positive cells in 10 random fields on one slide. IHC staining grade 0–2 was defined as low expression, and IHC staining grade 3–4 was defined as high expression.

### Cell Lines and Cell Culture

Human neuroblastoma cell lines SK-N-BE (2) and SK-N-AS were obtained from ATCC (Manassas, USA). All cell lines were cultured in a 1:1 mixture of Eagle's minimum essential medium and F12 medium (Gibco, USA) supplemented with 10% fetal bovine serum (Gemini, USA) in humid air at 37°C with 5% CO_2_.

### Knockdown and Overexpression of Prp19

Cells were plated in 6-, 12-, or 24-well-plates the day before transfection so that they achieved 30–50% confluence at the time of transfection. Cells were transfected with small interfering RNAs (siPrp19, siYAP, and negative control siNC) using RNAiMAX (ThermoFisher, USA) according to the manufacturer's instructions. siRNAs were synthetized by RiboBio (China); target sequences were as follows: Prp19-1 5′-GCCACTATCAGGATTTGGT-3′, Prp19-2 5′- GCCAAGTTCATCGCTTCAA-3′, and YAP 5′-GTAGCCAGTTACCAACACT-3′. Cells were incubated for 24 to 48 h at 37°C before harvesting cells for analyses.

A Prp19 overexpressing adenovirus with Flag and His tag was purchased from Vigenebio (China). Prp19 overexpression cell lines were constructed in SK-N-AS and SK-N-BE (2) cells.

### Cell Invasion Assay

Matrigel was added to the top chamber of Transwell chambers before the chambers were placed in an incubator for 4 h. Then, siRNA-treated cells or Prp19 overexpressing cells (1 × 10^5^ cells), having been washed with serum-free medium, were plated into chambers. After 36 h in 37°C, the cells were harvested and fixed with 4% paraformaldehyde for 20 min. Cells were then stained with 0.1% crystal violet staining for 10 min, and the Matrigel was wiped off with a cotton swab. Samples were observed under a microscope; 5–10 fields were randomly selected and photographed in each chamber.

### Wound-Healing Assay

SK-N-BE (2) and SK-N-AS cells were seeded into 6-well-plates; after 24 h, the cells were transfected or infected with siNC, siPrp19, siYAP, or Prp19 or control adenovirus. Forty-eight hours later, the cells reached 100% confluence, and the cell monolayer was scraped with a 200-μl pipette tip. The well was washed twice with serum-free medium (50% MEM and 50% F12) and then replenished with fresh serum-free medium. At 0 h and every 12 h after incubation, images were captured with a light microscope (LeicaCTR6000 microscope system) at x50 magnification and analyzed quantitatively by ImageJ (X64, v. 2.1.4).

### Protein Extraction and Western Blotting (WB)

WB was performed as previously described (23). Primary antibodies specific for Prp19 (ab126776, 1:1,000) and YAP (ab52771, 1:1,000) were purchased from Abcam; Primary antibodies specific for GAPDH (2118s, 1:2,000), cyclinD1 (2978s, 1:1,000), MMP9 (13667s, 1:1,000), E-cadherin (3195s, 1:1,000) were purchased from Cell Signaling Technology (Beverly, USA). A primary antibody specific for Actin was purchased from Yeasen (30101ES10, 1:5,000). Primary antibodies specific for CTGF (sc-101586, 1:200), FGF1 (sc-55520, 1:100) were purchased from Santa Cruz Biotechnology (USA). The results were quantitatively analyzed through Image J software (X64, v. 2.1.4).

### Polymerase Chain Reaction (PCR) and Quantitative Real Time PCR (qPCR)

Total RNA was isolated with TRIzol (Invitrogen, USA) and subjected to DNase I treatment prior to reverse transcription (Promega, USA). Reverse transcription reactions were performed using the reverse transcription kit from TakaRa (Tokyo, Japan). PCR was performed using the MAX PCR Master Mix (TakaRa, Japan), and the PCR products were subjected to agarose gel electrophoresis. qPCR was conducted to measure the levels of mRNAs using SYBR Green reagent from Yeasen (China). Primer sequences were as follows: *Prp19* forward 5′-GTGCCAAGTTCCCAACCAAGTGTT-3′, reverse 5′-AGCACAGTGGCTTTGTCTTGAAGC-3′; *YAP-1*: forward 5′-CCCGACTCCTTCTTCAAGC-3′, reverse 5′-TGTCCCAGGAGAAACAGCTC-3′; *YAP-2*: forward 5′-TTGTGCCAACTTGATTCAGC-3′, reverse 5′-TACATCCCGAGTGGGCTAAC-3′;*YAP-3*: forward 5′-CCTGCGTAGCCAGTTACCAA-3′, reverse 5′-CCATCTCATCCACACTGTTC-3′; *YAP-4*: forward 5′-TTGTCACCAAGCACAGAACC-3′, reverse 5′-TTCCTGTCCTGCAATGTCTG-3′; *GAPDH*: forward 5′-TCGACAGTCAGCCGCATCTTCTTT-3′, reverse 5′-GCCCAATACGACCAAATCCGTTGA-3′. The transcript levels were calculated and analyzed by the 2^−ΔΔCT^ method.

### RNA Sequencing Assay and Data Analysis

siPrp19 and siNC small interference RNA were transfected to SK-N-BE (2) and SK-N-AS cells for 48 h, and then 3 μg of total RNA per sample (6 samples per cell line) were extracted by TOIzol® reagent (Thermo Fisher scientific, American). Library preparation for transcriptome sequencing and clustering and sequencing were performed by Novogene Bioinformatics Technology Co., Ltd. (Beijing, China). Per sample, biological replicates were performed three times. Differential expression analysis of the two groups was performed using the DESeq2R package (1.16.1), and genes with an adjusted *P* < 0.05 found byDESeq2 were assigned as differentially expressed. Gene ontology (GO) enrichment analysis of differentially expressed genes was implemented by the cluster Profiler R package, in which gene length bias was corrected. GO terms with corrected value <0.05 were considered significantly enriched by differentially expressed genes. KEGG is a database resource for understanding high-level functions and utilities of the biological system from molecular-level information, especially large-scale molecular data sets generated by genome sequencing and other high-throughput experimental technologies (http://www.genome.jp/kegg/). We used the cluster Profiler R package to test the statistical enrichment of differential expression genes in KEGG pathways. Sequencing data have been deposited in the Gene Expression Omnibus database, and the accession codes of SK-N-BE (2) and SK-N-AS are GSE153398 and GSE153432.

### Neuroblastoma Data Set Analysis

In order to validate the conclusion we got from our clinical data, we analyzed the factors associated with the prognosis of neuroblastoma by the R2 Genomics Analysis and Visualization Platform (http://r2.amc.nl) using the following publicly available data sets: SEQC (GEO: GSE49710) and NRC (GEO: GSE85047). In addition, we also analyzed the differential expression of Prp19 among clinical stage, risk classification, *MYCN* status, and histology.

### Statistical Analysis

Statistical analysis was performed using SPSS 20.0 software. The Mann-Whitney U rank sum test was used for the non-normally distributed variables. The different expression of Prp19 among genders, ages, bone marrow infiltration, clinical stage (INSS), tumor pathological diagnosis, Shimada pathological classification, and risk classification were analyzed by chi-square test. Multivariate Cox regression analysis was performed on the effects of Prp19, clinical stage, and age at diagnosis on survival in children. Survival curves between different expression groups of Prp19 were delineated using Kaplan–Meier curves, and differential analysis of survival curves was performed using log-rank test. Values of *p* < 0.05 were considered statistically significant.

## Results

### Differential Expression of Prp19 Is Associated With Clinical Characteristics in Neuroblastoma

**Figure 1:**
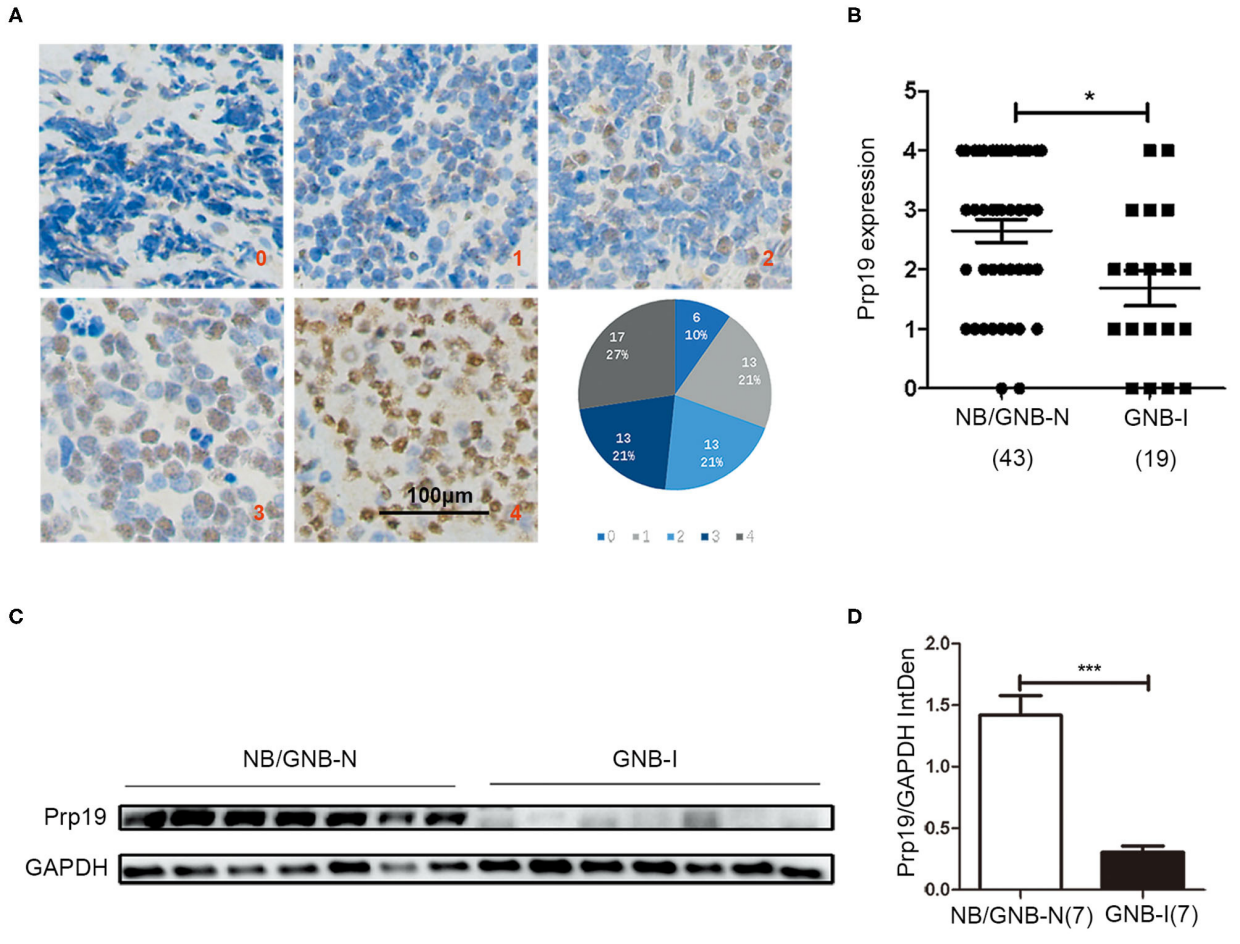
Expression and roles of Prp19 in neuroblastoma. **(A)** Representative images of different levels (0–4) of IHC staining of Prp19 and the proportions of five levels. Scale bar: 100 μm. **(B)** Prp19 expression between NB/GNB-N and GNB-I in TMA. The data were analyzed by Mann-Whitney *U*-test (*n* = 62). **(C,D)** Proteins of 7 NB/GNB-N and 7 GNB-I tumor tissue proteins extracted for immunoblotting to detect Prp19 expression, and Image J was used to quantify protein bands. The data in **(D)** were analyzed by Mann-Whitney *U*-test (*n* = 14). *p < 0.05 and ***p < 0.001.

We first performed IHC analysis of Prp19 expression on a TMA consisting of 62 samples from patients diagnosed with neuroblastoma, including 43 NB/GNB-N and 19 GNB-I cases. Prp19 was mainly expressed in the cell nucleus and differentially expressed in neuroblastoma patients with different pathological types; the expression of Prp19 was as follows: grade 0 (10%), grade 1 (21%), grade 2 (21%), grade 3 (21%), and grade 4 (27%) (Figure 1A). Prp19 expression in patients with NB/GNB-N was significantly higher than that in patients with GNB-I (*p* = 0.016; Figure 1B). Western blot analysis of 14 frozen fresh samples further confirmed that the expression of Prp19 was significantly higher in patients with NB/GNB-N compared with patients with GNB-I (*p* = 0.006; Figures 1C,D). This indicates that Prp19 shows differential expression in different pathological types of neuroblastoma.

We next examined whether expression of Prp19 had clinical implications in our cohort. As shown in Table 1, high expression of Prp19 was significantly associated with bone marrow metastasis, NB/GNB-N, unfavorable histologic and high risk (*p* = 0.009, 0.021, 0.022, and 0.023, respectively); however, there were no associations with age at diagnosis and clinical stage in our TMA, possibly due to insufficient sample size. To further confirm the clinical implications of Prp19 in human neuroblastoma, we analyzed gene expression profiles from two cohorts of neuroblastoma primary tumors with larger sample sizes (SEQC: GSE62564 and NRC: GSE85047). The expression of Prp19 was positively correlated with clinical stage (*p* < 0.001; Table 2), and more interestingly, Prp19 expression increased with clinical stage from stage I to stage IV, but significantly decreased in stage IVs (Supplementary Figures 1A,C). Furthermore, Prp19 was significantly overexpressed in children diagnosed at more than 18 months (*p* < 0.001) and high-risk cases (*p* < 0.001; Table 2 and Supplementary Table 1; Supplementary Figures 1B,D,E). In addition, patients with *MYCN* amplification also tended to have higher Prp19 expression (*p* < 0.001; Table 2 and Supplementary Table 1; Supplementary Figures 1F,G). Together, these results indicate that Prp19 shows differential expression in neuroblastoma tissues and higher Prp19 expression is associated with poor clinical characteristics in neuroblastoma.

**Table 1:** The association between Prp19 expression with clinical pathologic characteristics in the TMA cohort.

**Clinical and pathological characteristics**	**Total**	**Prp19**		**χ^2^**	***P***
		**High**	**Low**		
**Gender**					
Male	35	14	21	2.264	0.132
Female	27	16	11		
**Age at diagnosis**					
≥18 months	42	21	21	0.136	0.713
<18 months	20	9	11		
**Bone marrow metastasis**					
Positive	21	15	6	6.751	0.009
Negative	41	15	26		
**Clinical stages**					
I–II IV–S	25	10	15	1.180	0.277
III–IV	37	20	17		
**Diagnostic category**					
NB and GNB-N	43	25	18	5.344	0.021
GNB-I	19	5	14		
**Shimada pathologic type**					
FH	30	10	20	5.274	0.022
UFH	32	20	12		
**Risk classification**					
Low and					
Intermediate	34	12	22	5.168	0.023
High	28	18	10		

*NB, neuroblastoma; GNB-N, ganglioneuroblastoma-nodular; GNB-I, ganglioneuroblastoma-intermixed; FH, favorable histology; UFH, unfavorable histology*.

**Table 2:** Correlation analysis between clinical characteristics and expression of Prp19 in the SEQC data set.

**Covariates**	**Total**	**Prp19**		**χ^**2**^**	***P***
		**High**	**Low**		
**Age at diagnosis**					
≥18 months	198	94	104		
<18 months	300	45	255	62.517	<0.001
**Clinical stages**					
I–II IV–S	252	18	234		
III–IV	246	121	125	109.362	<0.001
**Risk**					
Low risk	322	33	289		
High risk	176	106	70	498.000	<0.001
***MYCN* state**					
Amplification	92	50	42		
Non-amplification	401	87	314	39.758	<0.001

### High Expression of Prp19 Is a Potential Poor Prognostic Factor in Children With Neuroblastoma

**Figure 2:**
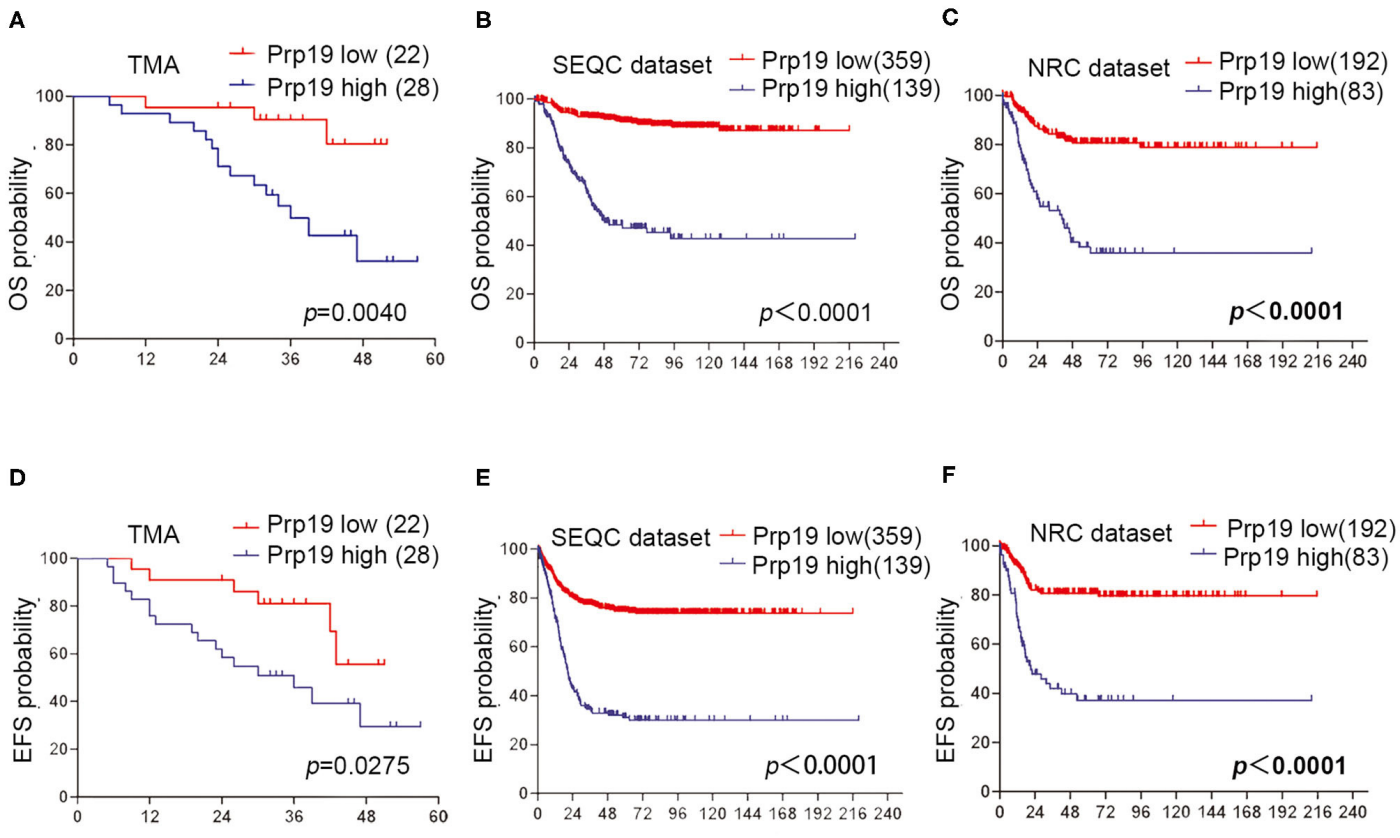
Prp19's prognostic value in TMA cohort and data sets. **(A,D)** Kaplan-Meier analysis of OS and EFS for the TMA cohort based on Prp19 expression with the log-rank test *P*-value indicated (*n* = 50). **(B,E)** Kaplan-Meier analysis of OS and EFS for the SEQC data set based on Prp19 expression with the log-rank test *p* value indicated (*n* = 498). **(C,F)** Kaplan-Meier analysis of OS and EFS for the NRC data set based on Prp19 expression with the log-rank test *p*-value indicated (*n* = 275).

We next investigated if Prp19 had an influence on the prognosis of children with neuroblastoma. Out of the 62 patients from the TMA cohort, 50 cases were successfully followed up with a follow-up completion rate of 80.6%. The median follow-up time was 34 months, and the longest and shortest follow-up periods were 62 and 6 months, respectively. We examined OS and EFS by Kaplan-Meier curves in the TMA cohort and compared the survival curves by log-rank test. Patients with low Prp19 expression had a better OS (*p* = 0.0040) and EFS (*p* = 0.0275; Figures 2A,D). In addition, analyses of the SEQC and NRC data sets revealed similar results, and both the *p*-value of all OS and EFS rates were <0.0001 (Figures 2B,C,E,F). In the TMA cohort, univariate analysis found that high expression of Prp19 was a risk factor both for OS (*p* = 0.010; HR = 5.189) and EFS (*p* = 0.045; HR = 2.644) in children with neuroblastoma (Table 3). Multivariate analysis also found that patients with high Prp19 expression had a worse OS (*p* = 0.027; HR = 4.118) although there was no statistically significant difference in the EFS (*p* = 0.174; HR = 1.954). Clinical stage, as a recognized prognostic risk factor, was also associated with poor OS (*p* = 0.016; HR = 12.345) and EFS (*p* = 0.003; HR = 9.267). However, age at diagnosis did not show an effect on OS and EFS in children with neuroblastoma (Table 4). Age at diagnosis is a known risk factor affecting the prognosis of children with neuroblastoma (3). On account of this, we doubted that the negative result from Table 4 was limited by the number of the TMA cohort. To further validate our results, we performed the analyses in the SEQC and NRC databases. Univariate and multivariate analyses show that high Prp19 expression, high clinical stage, and age over 18 months at diagnosis are associated with poor OS and EFS in the SEQC data set (Supplementary Tables 2, 3). In the NRC data set, OS and EFS are associated with Prp19 expression and clinical stage, but age at diagnosis, even with an impact on OS, has no effect on EFS (Supplementary Tables 4, 5). Taken together, our analyses indicate that Prp19 is a potential prognostic marker in neuroblastoma.

**Table 3:** Univariate analyses in the TMA cohort.

**Covariates**	**OS**		**EFS**	
	**HR (95%CI)**	***P***	**HR (95%CI)**	***P***
**Prp19 expression** (high vs. low)	5.189 (1.487–18.109)	0.010	2.644 (1.023–6.836)	0.045

**Table 4:** Multivariable analyses in the TMA cohort.

**Covariates**	**OS**		**EFS**	
	**HR (95%CI)**	***P***	**HR (95%CI)**	***P***
**Prp19 expression** (high vs. low)	4.118 (1.171–14.480)	0.027	1.954 (0.744–5.136)	0.174
**Clinical stages** (I–II IV–S vs. III–IV)	12.345 (1.613–94.489)	0.016	9.267 (2.114–40.624)	0.003
**Age at diagnosis** (< >18 months)	0.980 (0.351–2.740)	0.969	1.755 (0.667–4.616)	0.255

### Prp19 Promotes Neuroblastoma Cell Invasion and Migration

**Figure 3:**
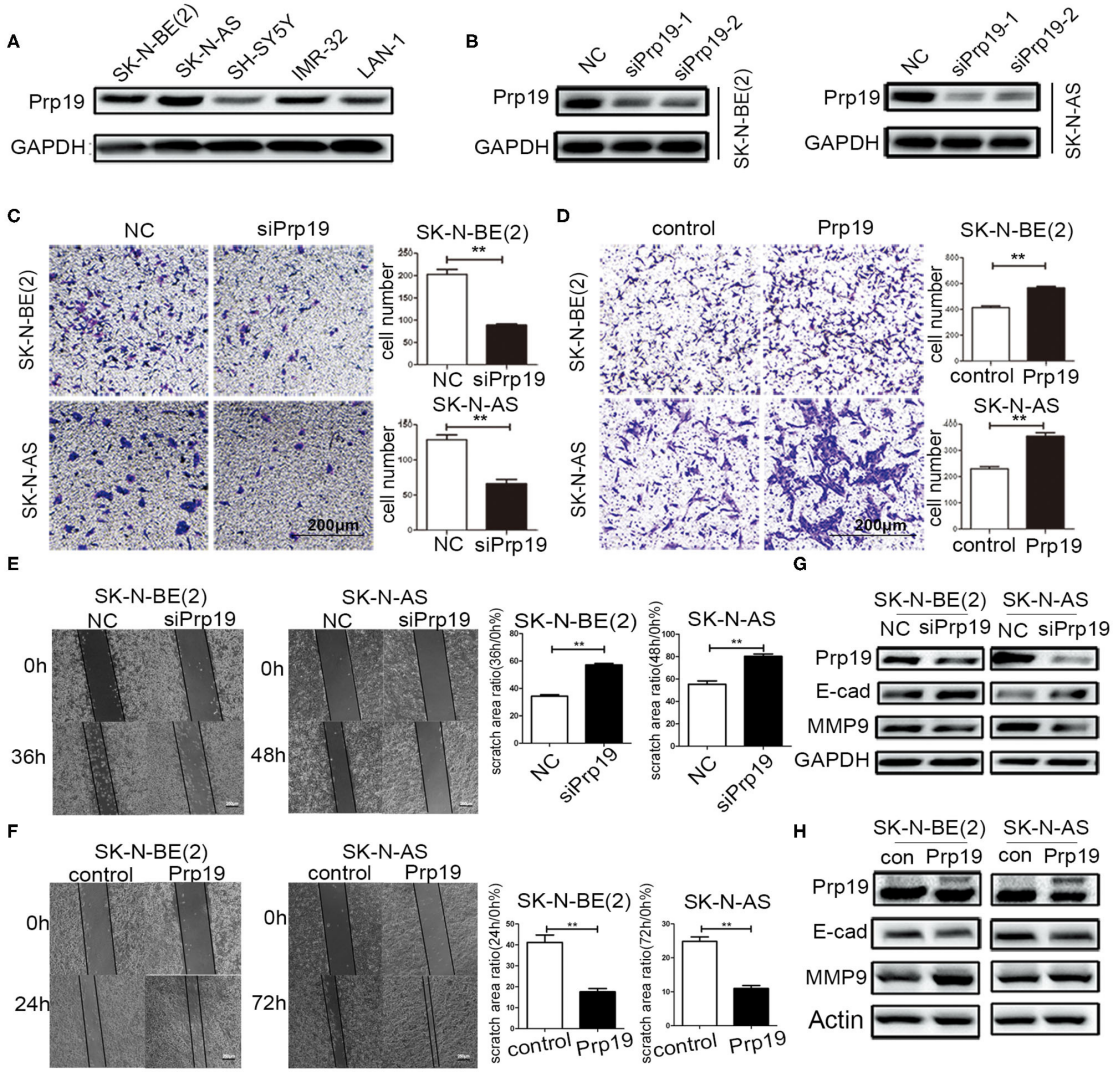
Effects of Prp19 on cell biological behavior of neuroblastoma cells. **(A)** WB detected the expression levels of Prp19 in neuroblastoma cell lines SK-N-BE (2), SK-N-AS, SH-SY5Y, IMR-32, and LAN1, and two cell lines [SK-N-BE (2) and SK-N-AS] with higher expression of Prp19 were selected for subsequent experiments. **(B)** Prp19 was knocked down in SK-N-BE (2) and SK-N-AS using siRNA. The first siRNA was selected for subsequent experiments according to knockdown efficiency detected by WB. **(C,D)** Transwell array tested the invasive ability of SK-N-BE (2) and SK-N-AS between siPrp19 and siNC or between overexpression of Prp19 and control, and the number of cells crossing the bottom of the chamber were analyzed by GraphPad Prism 5. Scale bar: 200 μm *p* = 0.0022, respectively. **(E,F)** The cell migration ability was estimated using a wound-healing assay. The images were captured at indicated time after wounding (magnification: 50×; scale bar: 250 μm) *p* = 0.0022, respectively. **(G,H)** WB tested the expression of Prp19, E-cad, and MMP9 in Prp19 downregulation or overexpression cells. *p < 0.05 and **p < 0.01.

Then, the function of Prp19 at the cellular level was explored. First, the expression level of Prp19 in different neuroblastoma cell lines [SK-N-BE (2), SK-N-AS, SH-SY5Y, IMR32, and LAN1] was assessed, and the silencing efficiency of siRNA targeting Prp19 was examined (Figures 3A,B). Two cell lines [SK-N-BE (2) and SK-N-AS] with higher expression of Prp19 and the first siRNA were selected for the following assays. We next explored whether Prp19 influences migration or invasion of neuroblastoma cells. After 48 h silencing of Prp19 in SK-N-BE (2) and SK-N-AS cells, transwell assays were performed. As shown in Figures 3C,D, knockdown of Prp19 impaired the invasive ability of both SK-N-BE (2) and SK-N-AS cells, and upregulating Prp19 enhanced the invasive capacity of two cell lines. In addition, the wound-healing assay also showed increased or decreased migratory ability in Prp19 overexpressing cells or Prp19 silenced cells (Figures 3E,F). EMT-related molecules, such as E-cadherin and matrix metalloproteinase 9 (MMP9), were altered upon Prp19 expression; we observed increased E-cadherin and decreased MMP9 after Prp19 downregulation with opposite effects in Prp19 overexpression cells (Figures 3G,H). These results demonstrate the role of Prp19 in neuroblastoma cell invasion, migration, and EMT.

### RNA-seq Identifies the Hippo/YAP Pathway as a Candidate Regulatory Target of Prp19

**Figure 4:**
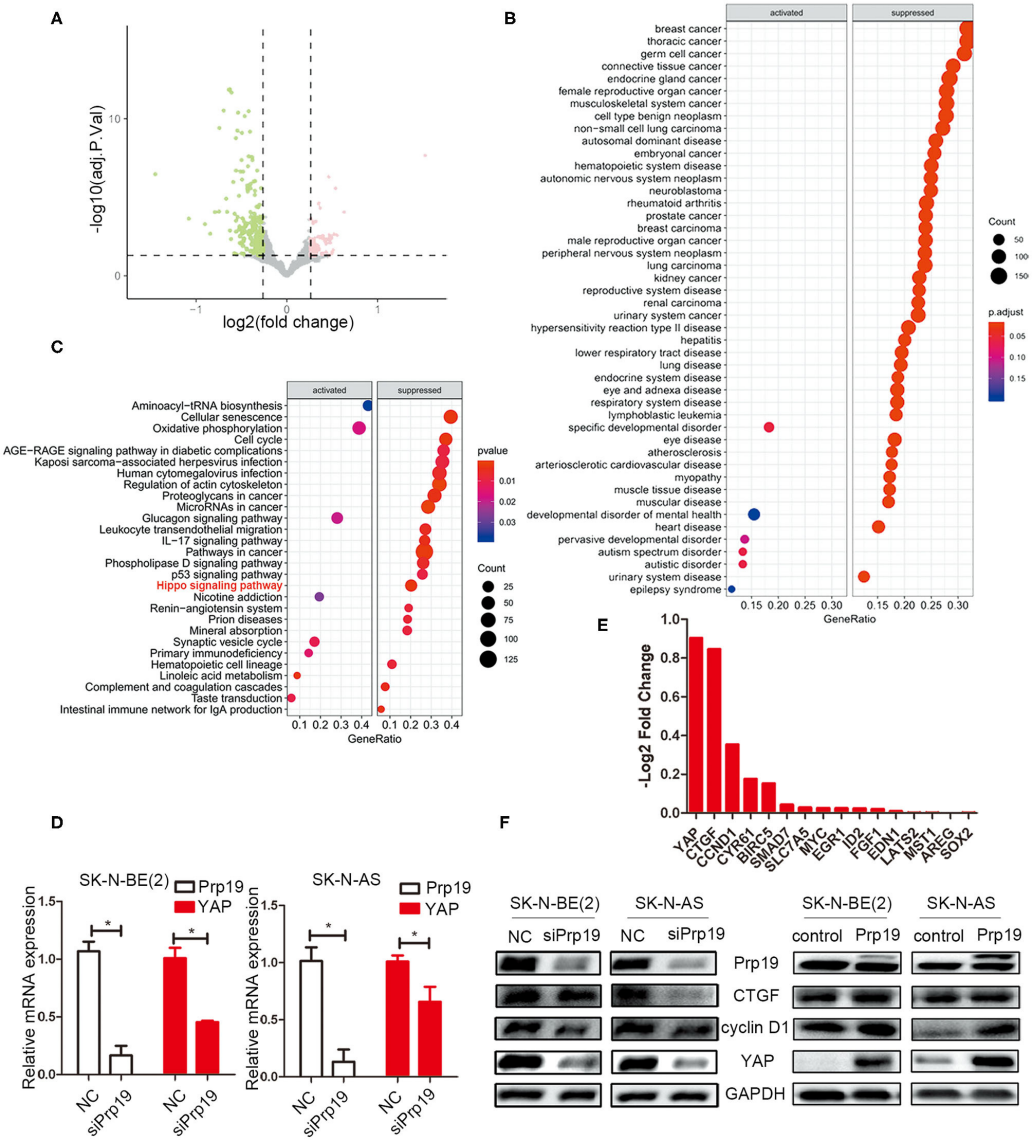
Bioinformatics analysis of RNA-seq data. SK-N-BE (2) cells were transfected with siPrp19 for 48 h, and then subjected to RNA sequencing. **(A)** Volcano plot showing distribution of differential expression genes Prp19 downregulation (|Fold of changes| >1.5, FDR <0.05). **(B)** Disease ontology (DO) analysis enriched the affected diseases after Prp19 knockdown, which were mainly concentrated in nervous system cancer. **(C)** KEGG enriched the affected pathways after Prp19 knockdown, indicating that the Hippo signaling pathway was suppressed. **(D)** PCR analyzed the YAP mRNA level after Prp19 knockdown in SK-N-BE (2) and SK-N-AS. **(E)** RNA-seq analyzed the mRNA level of YAP and its downstream genes after Prp19 knockdown. It indicated that YAP and its downstream genes decreased following Prp19 downregulation. **(F)** The protein level of YAP, cyclin D1, and CTGF after downregulation or overexpression of Prp19. *p < 0.05.

Given the change of invasion and migration observed in Prp19 knockdown and overexpressed cells, we speculated that Prp19 may affect specific cellular functions through regulating specific gene expression. To explore the mechanism by which Prp19 exerts its function in neuroblastoma, we performed RNA-seq analysis using SK-N-BE (2) and SK-N-AS cells transfected with siPrp19 compared with cells transfected with siNC. More than 400 genes were significantly regulated upon Prp19 downregulation (|fold change| >1.5, FDR <0.05; Figure 4A and Supplementary Figure 2A). Bioinformatic analysis shows that most of the enriched disease ontology (DO) catalogs and signaling pathways are suppressed upon Prp19 depletion. DO semantic and enrichment analysis reveal that the most highly enriched functional categories are related to cancer, including central nervous system cancer, autonomic nervous system neoplasm, and even neuroblastoma (adj. *p* < 0.05; Figure 4B and Supplementary Figure 2B). Results from KEGG analysis identified the Hippo-YAP pathway (Figure 4C and Supplementary Figure 2C), which has been shown to regulate multiple biological processes in various cancers but has not been indicated as downstream signaling pathway of Prp19. YAP, the key component of the Hippo-YAP signaling pathway, was significantly downregulated at both the mRNA and protein levels in cells silenced for Prp19 (Figures 4D,F) and was decreased in both the cytoplasm and nucleus (Supplementary Figure 3). Furthermore, the downstream genes of the Hippo-YAP pathway, including *CTGF* and *CCND1*, were also significantly decreased with Prp19 knockdown (Figure 4E and Supplementary Figure 2D). In addition, the protein levels of YAP, CTGF, and cyclin D1 were also decreased or increased upon Prp19 downregulation or overexpression, respectively (Figure 4F). Together these data, thus, suggest that Prp19 may be involved in regulating the Hippo-YAP pathway by regulating YAP level.

### Effect of Deletion of YAP on Cell Invasion and Migration

**Figure 5:**
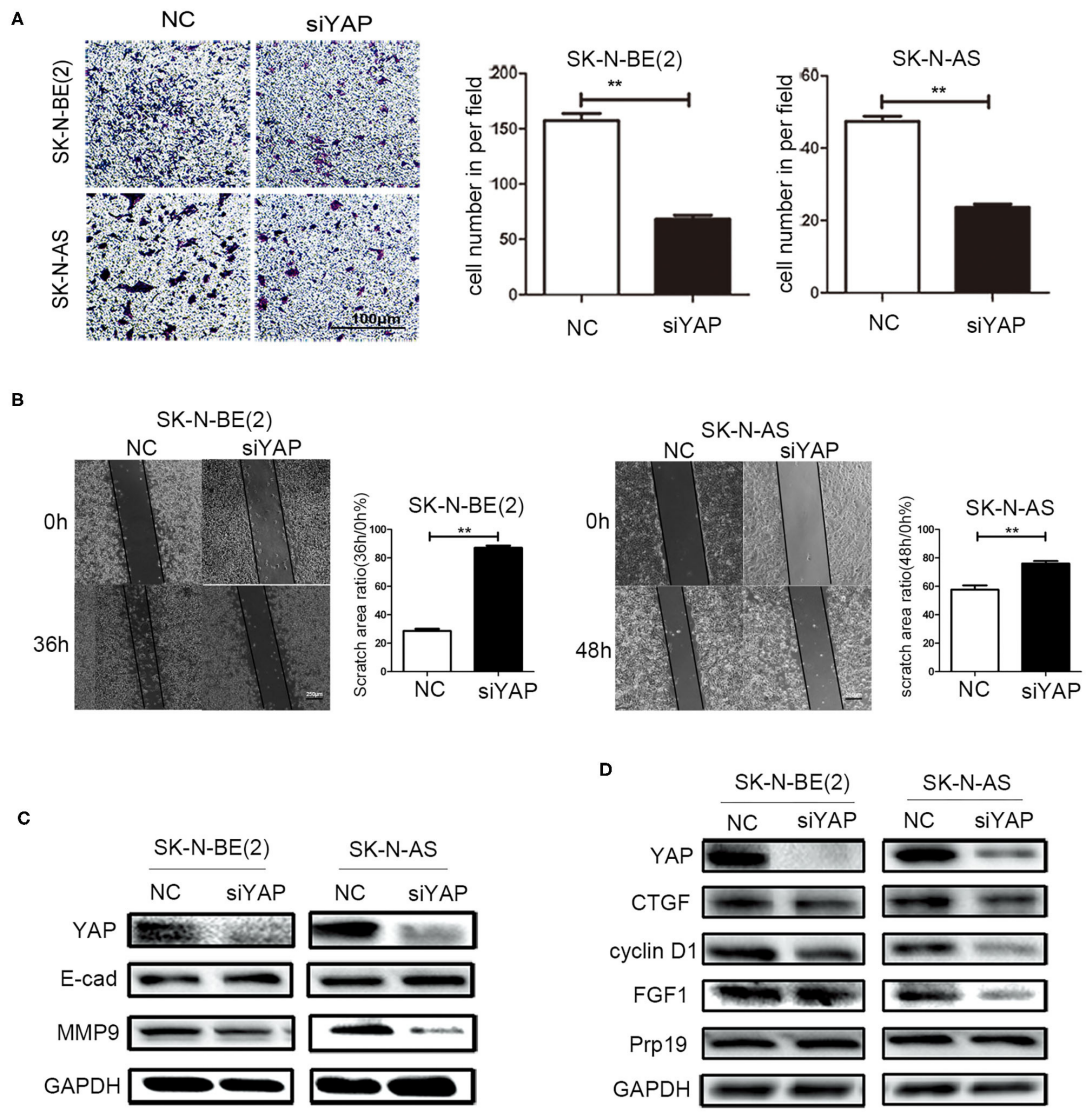
Effects of YAP on cell biological behavior of neuroblastoma cells. **(A)** Transwell array tested the invasive ability of SK-N-BE (2) and SK-N-AS between siYAP and siNC, and the number of cells crossing the bottom of the chamber were analyzed by GraphPad Prism 5. Scale bar: 100 μm *p* = 0.0079, respectively. **(B)** The cell migration ability was estimated using the wound-healing assay after YAP downregulation. The images were captured at the time shown on the graph after wounding (magnification: 50×; scale bar: 250 μm) *p* = 0.0022, respectively. **(C)** WB tested the expression of YAP, E-cad, and MMP9 after downregulation of YAP. **(D)** WB tested the expression of YAP, CTGF, cyclin D1, FGF1, and Prp19 after downregulation of YAP. *p < 0.05 and **p < 0.01.

To examine whether Prp19 affects invasion and migration of neuroblastoma cells via the Hippo-YAP pathway, we first examined whether silencing YAP resulted in similar changes of invasion and migration of neuroblastoma cells as with Prp19 silencing. As a result, as we expected, invasion and migration abilities were impaired in YAP downregulated cells (Figures 5A,B). YAP knockdown was also accompanied with upregulation of E-cadherin and downregulation of MMP9 (Figure 5C). Expressions of downstream target genes of YAP were also downregulated after YAP knockdown, but Prp19 expression was not affected (Figure 5D). These results demonstrate that YAP knockdown causes almost same changes as Prp19 knockdown, implying that Prp19 may affect cell invasion and migration by regulating YAP expression.

### Prp19 and YAP Are Involved in Neuroblastoma Metastasis

**Figure 6:**
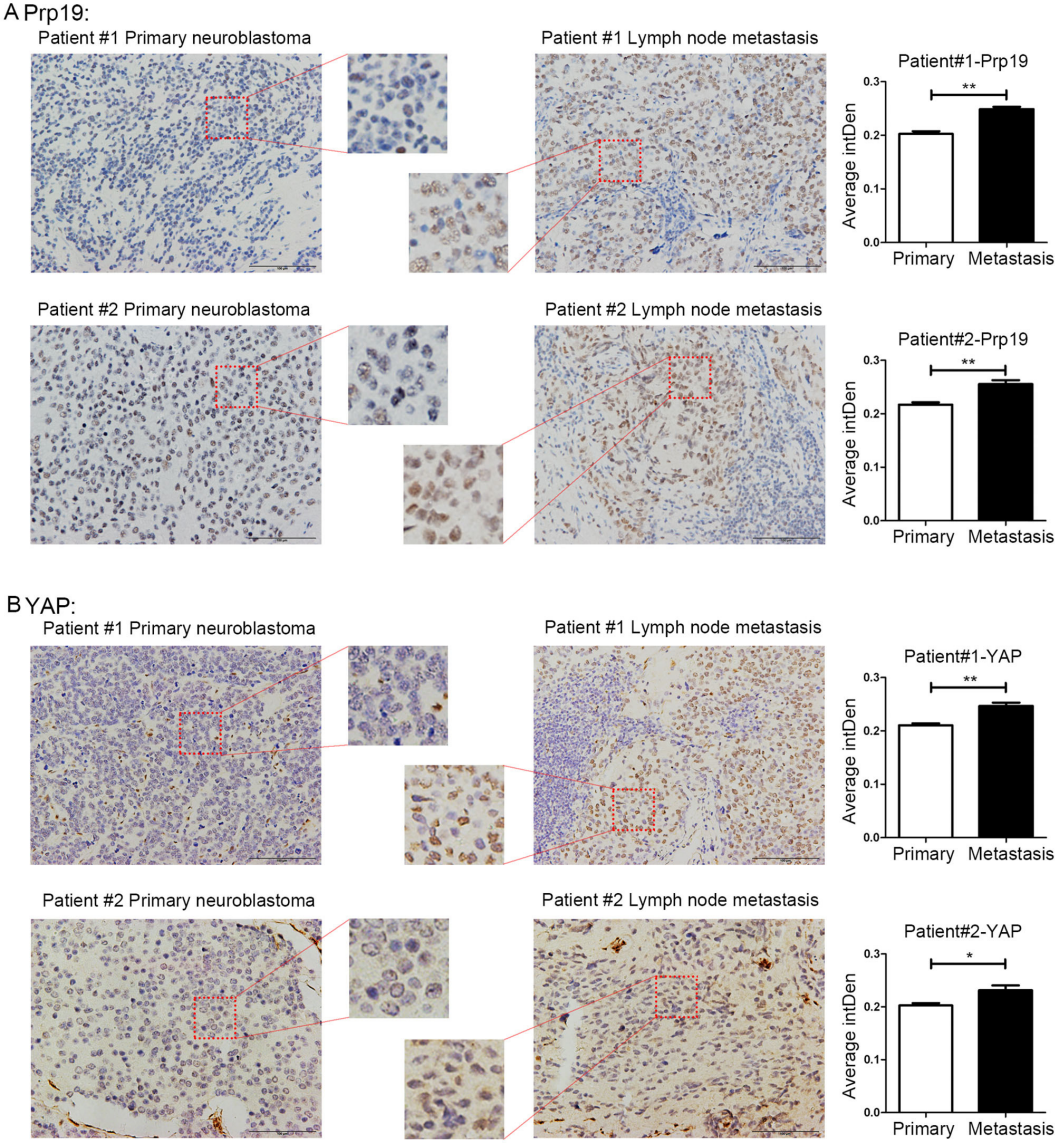
Expression of Prp19 and YAP are higher in metastatic lymph nodes than primary sites. **(A)** The expression of Prp19 in metastatic lymph nodes of patients 1 and 2 is higher than that in primary tumor. **(B)** The expression of Prp19 in metastatic lymph nodes of patients 1 and 2 is higher than that in primary tumor. The average intDen in each group was analyzed by Mann-Whitney *U*-test (*n* = 6 per group). *p < 0.05 and **p < 0.01.

Because the enhancement of invasion and migration ability is closely related to metastasis, we speculated that Prp19 may play a role in tumor metastasis. Four pairs of neuroblastoma *in situ* tumor and metastatic lymph node tissues for the IHC experiment were collected. Among the 4 pairs of samples, Prp19 and YAP expression in two lymph node samples were higher than their corresponding tissues (Figure 6). The other 2 pairs of samples had received chemotherapy before we obtained them, so they did not show consistent results (data not shown). This result demonstrated Prp19 and YAP participate in tumor metastasis.

### Prp19 Is Required for Efficient RNA Processing of YAP mRNA

**Figure 7:**
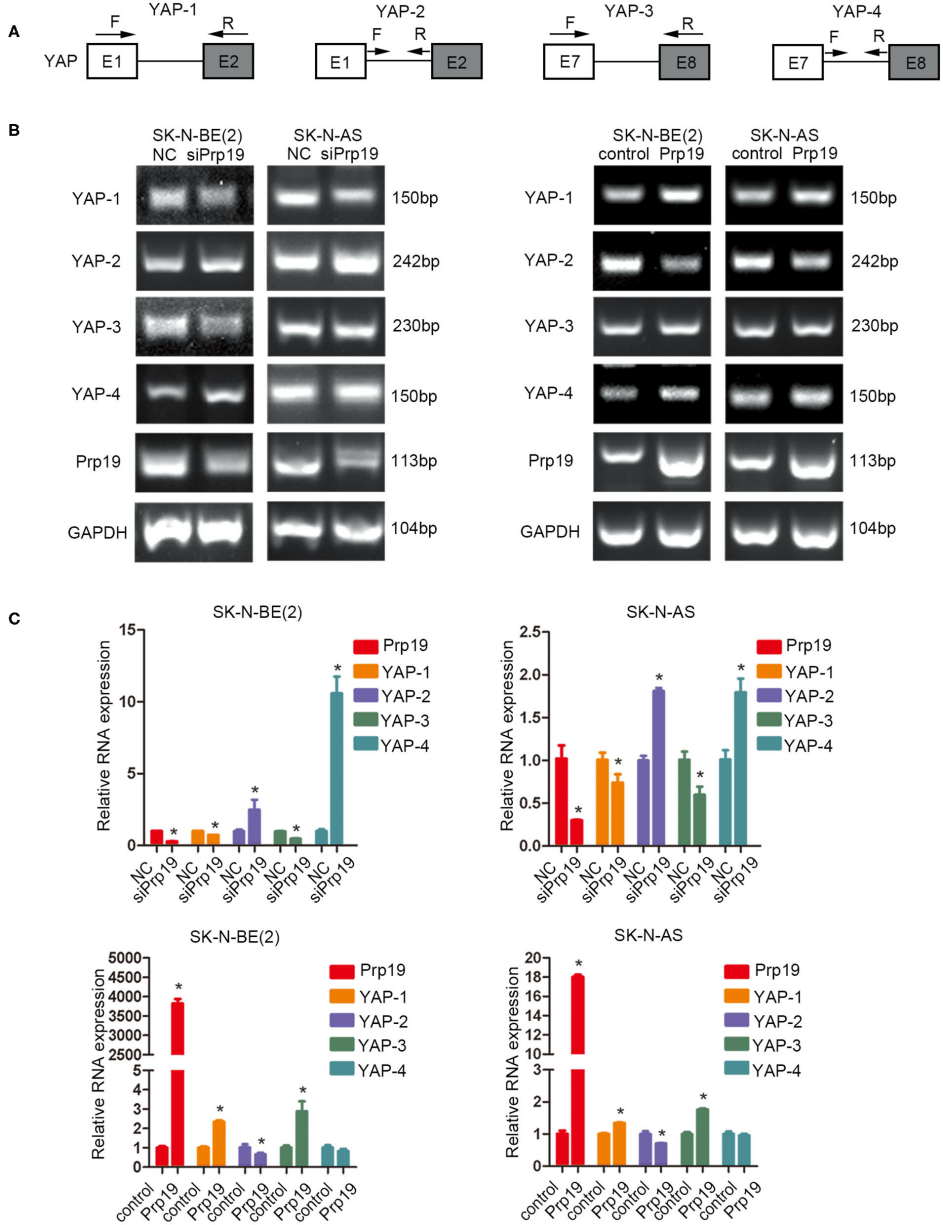
Prp19 is required for efficient intron removal of YAP. **(A)** A schematic diagram of exons and introns of the YAP gene and the primer sets designed for PCR as well as the RT-PCR shown in **(B,C)**. **(B)** PCR analysis using primer sets shown in **(A)** to compare splicing efficiencies of YAP introns splicing in Prp19 siRNA-transfected and Prp19 overexpression cells. **(C)** qPCR analysis using primer sets shown in **(A)** to compare splicing efficiencies of YAP introns splicing in Prp19 siRNA-transfected and Prp19 overexpression cells. *p < 0.05.

Previous studies establish a critical role for Prp19 in regulating pre-RNA splicing. Our results show that Prp19 affects YAP gene levels, and we, therefore, examined the possibility that downregulation of YAP expression after Prp19 knockdown might be due to inefficient pre-mRNA splicing. To explore this possibility, we designed a series of PCR primer pairs targeting two neighboring constitutive exons or the interior of a single intron of the YAP gene as shown in Figure 7A. PCR agarose gel electrophoresis analyses found YAP intron retention (YAP-2 and YAP-4) and mature mRNA reduction (YAP-1 and YAP-3) in SK-N-BE (2) and SK-N-AS cells upon Prp19 knockdown, and opposite changes were found in Prp19 overexpression cells in addition to YAP-4 (Figure 7B). We verified the results with qPCR and obtained similar results. The relative RNA levels of YAP-1 and YAP-3, representing mature YAP RNA levels, decreased, and YAP-2 and YAP-4, representing immature YAP RNA levels, increased in Prp19 knockdown cells. In Prp19 overexpression cells, the relative RNA levels of YAP-1 and YAP-3 increased, and relative RNA levels of YAP-2 decreased while the relative RNA level of YAP-4 was unaffected (Figure 7C). These results demonstrated that Prp19 mediated YAP RNA splicing to regulate YAP abundance.

## Discussion

The International Neuroblastoma Pathology Classification (INPC) divides neuroblastoma into four subtypes: neuroblastoma, ganglioneuroblastoma-nodular (GNB-N), ganglioneuroblastoma-intermixed (GNB-I), and ganglioneuroma (GN) (24). Both GNB-I and GN have favorable clinical and biological characteristics, and the long-term survival rate exceeds 90% while neuroblastoma and GNB-N have relatively malignant clinical and biological characteristics with a long-term survival rate <80% (25). A recent study even indicated that GNB-I and GN patients only need regular follow-up rather than surgery first (26). Furthermore, in the current risk classification system, most GNB-I and GN are classified as low risk, whereas most neuroblastoma and GNB-N are classified as intermediate or high risk, which always accompany long-distance metastasis (3). Therefore, there may exist a different biological molecular distribution between neuroblastoma/GNB-N and GNB-I/GN that drive neuroblastoma/GNB-N possessing malignant biological behavior, for example, metastasis. In recent years, several studies have pointed out that molecular markers, for example, gene expression classification, can accurately affect the biological behavior, predicting outcomes for children with neuroblastoma (27–30). Revealing the molecular mechanisms of high-risk neuroblastoma may provide clues for target therapy, thereby improving the prognosis of children with high-risk neuroblastoma.

Our study reveals differential expression of Prp19 in different pathological types with higher Prp19 expression in neuroblastoma and GNB-N compared with GNB-I. This is consistent with several studies that show higher Prp19 expression in colon, laryngeal, and hepatocellular carcinoma tissues compared with normal tissues (13). In the SEQC and NRC databases, Prp19 expression was positively correlated with bone marrow metastasis, histological type, Shimada pathologic type and risk grade, age at diagnosis and clinical stage, and overexpression of Prp19 had a worse OS and EFS, which was also consistent with a previous study (13, 14). These data suggest that Prp19 may be one of the factors that results in differences in tumor biological behavior and prognosis in neuroblastoma patients.

The malignant biological behavior of tumors usually includes uncontrolled proliferation, metastasis, and multidrug resistance. In our clinical data, we noticed that Prp19 expression was positively correlated with bone marrow metastasis. Therefore, metastasis may be one of the factors by which Prp19 causes poor prognosis of neuroblastoma. EMT has been associated with various tumor functions, including tumor initiation, tumor stemness, tumor cell migration, intravasation to the blood, metastasis, and resistance to therapy (31, 32). The characteristic of EMT is the cell conversion from the epithelial-like profile (marked with E-cadherin and β-catenin) to the mesenchymal phenotype (marked with N-cadherin and MMP9) concomitant with the detachment of cells from intercellular adhesion and enhanced motility (32). A few studies have examined the involvement of Prp19 in EMT and metastasis of cancer. There is only one study showing that Prp19 promotes invasion, migration, and EMT in hepatocellular carcinoma cells (13). Here, we also found that downregulation of Prp19 inhibited invasion and migration of neuroblastoma cells and reversed EMT with an upregulation of E-cadherin and downregulation of MMP9, and overexpression of Prp19 had the opposite results. Abnormal activation of EMT not only results in the increased invasion and migration of cancer cells, but also triggers distant metastasis in diverse cancers (33, 34). Collectively, our findings identify the role of Prp19 in promoting cell invasion, migration, and EMT in neuroblastoma, and this means that Prp19 has the potential to promote tumor metastasis.

Given the pleiotropic changes observed in Prp19 knockdown cells, we hypothesized that Prp19 might take part in the regulation of certain genes, thereby regulating the expression of multiple genes. In order to seek a downstream target or pathway of Prp19, RNA-seq analysis was performed on neuroblastoma cells transiently transfected with Prp19 siRNA. Among the downregulated genes, it is noteworthy that the YAP gene changed by more than 0.9-log2fold when Prp19 was lowered by 1.0-log2fold. To the best of our knowledge, this is the first report suggesting that Prp19 may regulate the Hippo-YAP pathway in neuroblastoma cells. The Hippo-YAP pathway is a highly conserved signaling pathway regulating the regulation of organ size, cell proliferation, invasion, and apoptosis (17, 35). Many studies have shown that YAP, one of the key downstream terminal effectors of the Hippo pathway (36), promotes proliferation and metastasis of cancer cells (18, 37–39), and can also be regulated by other protein (21). Further analysis of changes in disease types and cellular pathways associated with downregulation of Prp19 found that the most highly enriched functional categories were related to cancer and that the Hippo-YAP pathway was inhibited. Results also imply that many protein and mRNA expressions were downregulated downstream of YAP. In addition, silencing YAP led to decreased invasive and migratory ability and reversal of EMT in neuroblastoma cells, similar to the effects of siPrp19. These results indicate that Prp19 might mediate tumor cell invasion and migration by regulating YAP.

The above results suggest that both Prp19 and YAP have the potential to promote tumor metastasis because they can promote tumor migration, invasion, and EMT transformation. Recent literature also has shown that YAP can promote tumor metastasis to lymph nodes and is highly expressed in metastatic lymph nodes (18). On this basis, our results reveal that the expression of Prp19 and YAP in neuroblastoma metastatic lymph nodes is significantly higher than that in *in situ* tumors. From the above results, we can see that Prp19 can affect the expression of YAP, but not the opposite. Therefore, it can be speculated that Prp19 promotes YAP expression and further promotes lymph node metastasis of the tumor though further experiments need to be done to verify.

The next point is how Prp19 regulates YAP expression. As far as we know, the best-described function of Prp19 is its role in splicing (40–42), a critical step in gene expression that involves the removal of introns with specificity and precision. As a pivotal step in eukaryotic gene regulation, splicing enables excision of introns from pre-mRNA and the generation of protein-coding transcripts, which takes place on the spliceosome, a dynamic macromolecular complex. Prp19 associates with the spliceosome concomitantly with or immediately after dissociation of U4, finally leading to spliceosomal activation and pre-mRNA splicing (8). We designed a series of PCR primer pairs targeting two neighboring constitutive exons, reflecting the mature mRNA, and the interior of a single intron of the YAP gene, reflecting the immature pre-mRNA. Results show that the mature mRNA of YAP is decreased with the decrease of Prp19 and increased with the increase of Prp19 except for YAP-4 in the Prp19 overexpression group. As for the YAP-4 group, it may be hypothesized that once Prp19 is overexpressed, the expression of YAP increased dramatically, so there was also a higher immature pre-mRNA of YAP. Taken together, these findings demonstrate that Prp19 controls YAP abundance by coordinated regulation of RNA splicing.

In conclusion, our study showed that Prp19 is positively correlated with various adverse clinical pathological parameters and could serve as a potential prognostic marker indicating a poor clinical prognosis in neuroblastoma. Moreover, Prp19 promotes metastasis of neuroblastoma cells through controlling the level of YAP by RNA splicing.

## Data Availability Statement

The original contributions presented in the study are publicly available. This data can be found in Bioproject GSE153398 and GSE153432.

## Ethics Statement

The studies involving human participants were reviewed and approved by Ethics Committee of Xin Hua Hospital Affiliated to Shanghai Jiao Tong University School of Medicine. Written informed consent to participate in this study was provided by the participants' legal guardian/next of kin.

## Author Contributions

YC carried out the studies, participated in the study design, statistical analysis, and drafted the manuscript. KC performed cell culture and participated in all *in vitro* experiments. KC, CC, and QC performed proteomics analysis and analyzed the RNA-Seq data. YX and GX participated in the histological examination of tissue samples and shRNA interference assay. ZW and YW conceived the study, participated in its design and coordination, and helped to draft the manuscript. All authors read and approved the final manuscript.

## Conflict of Interest

The authors declare that the research was conducted in the absence of any commercial or financial relationships that could be construed as a potential conflict of interest.
