# Supplementary material for: Prp19 Is an Independent Prognostic Marker and Promotes Neuroblastoma Metastasis by Regulating the Hippo-YAP Signaling Pathway
Source: Front Oncol. 2020 Nov 2;10:575366. doi: 10.3389/fonc.2020.575366 (PMC7667276; doi:10.3389/fonc.2020.575366)
Supplement: Supplementary file 1 [file Table_1.DOCX]

**Supplementary Material**

1. **Supplementary Figures and Tables**

**1.1 Supplementary Figures**


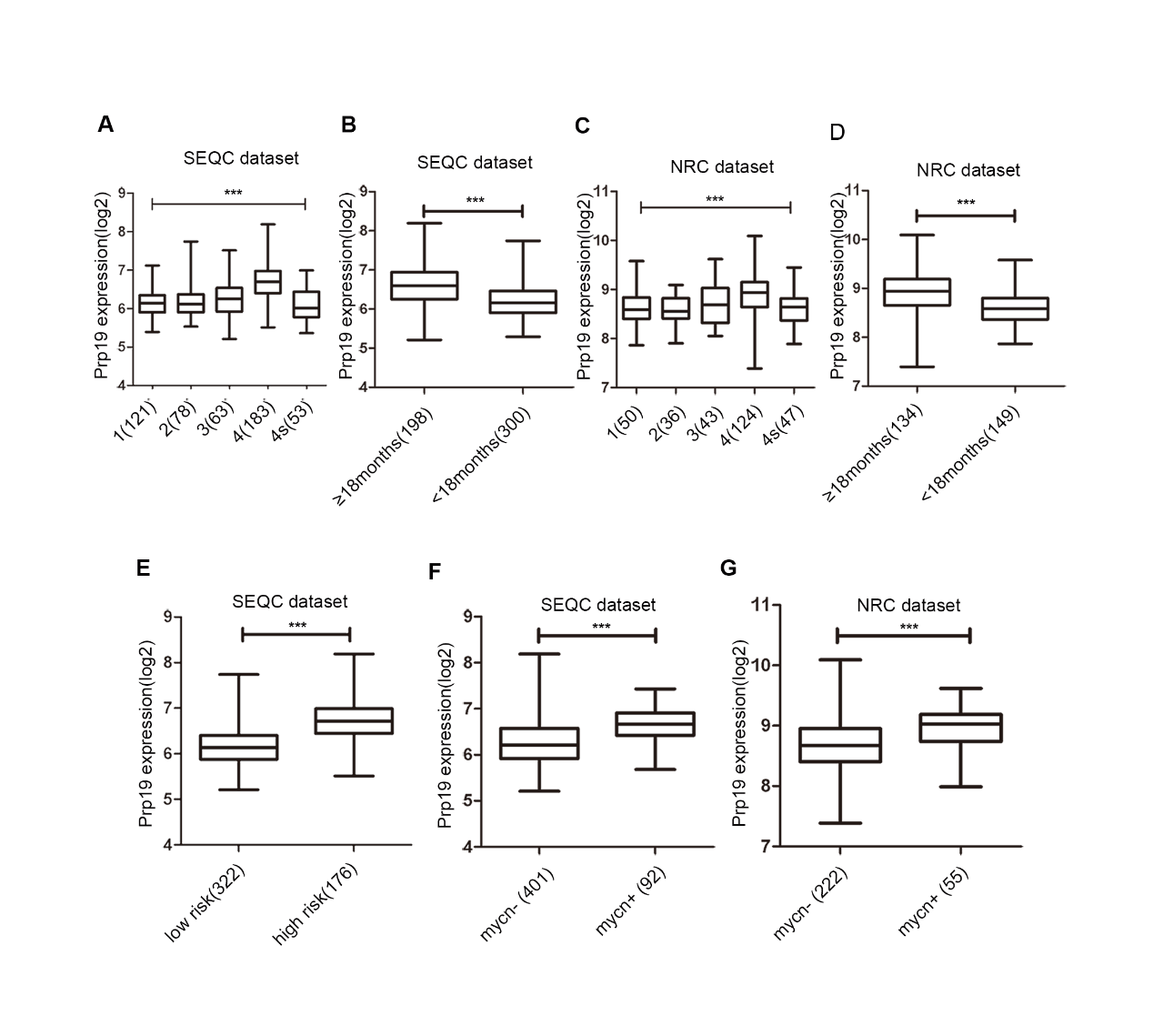


**Supplementary Fi****gure 1.** Relationship between Prp19 and clinical information in the SEQC and NRC neuroblastoma databases. **(A,C)** In dataset SEQC and NRC, box plots of Prp19 expression in different clinical stages (1-4 and 4s). **(B,D)** In dataset SEQC and

NRC, age at diagnosis more than 18 months have higher Prp19 expression. **(E)** Box plot of Prp19 expression between high-risk and low-risk groups in SEQC. **(F,G)** In dataset SEQC and NRC, box plots of differential expression of Prp19 between *mycn*

amplified and non-amplified. * *p* < 0.05, ** *p*< 0.01 and ****p* < 0.001.


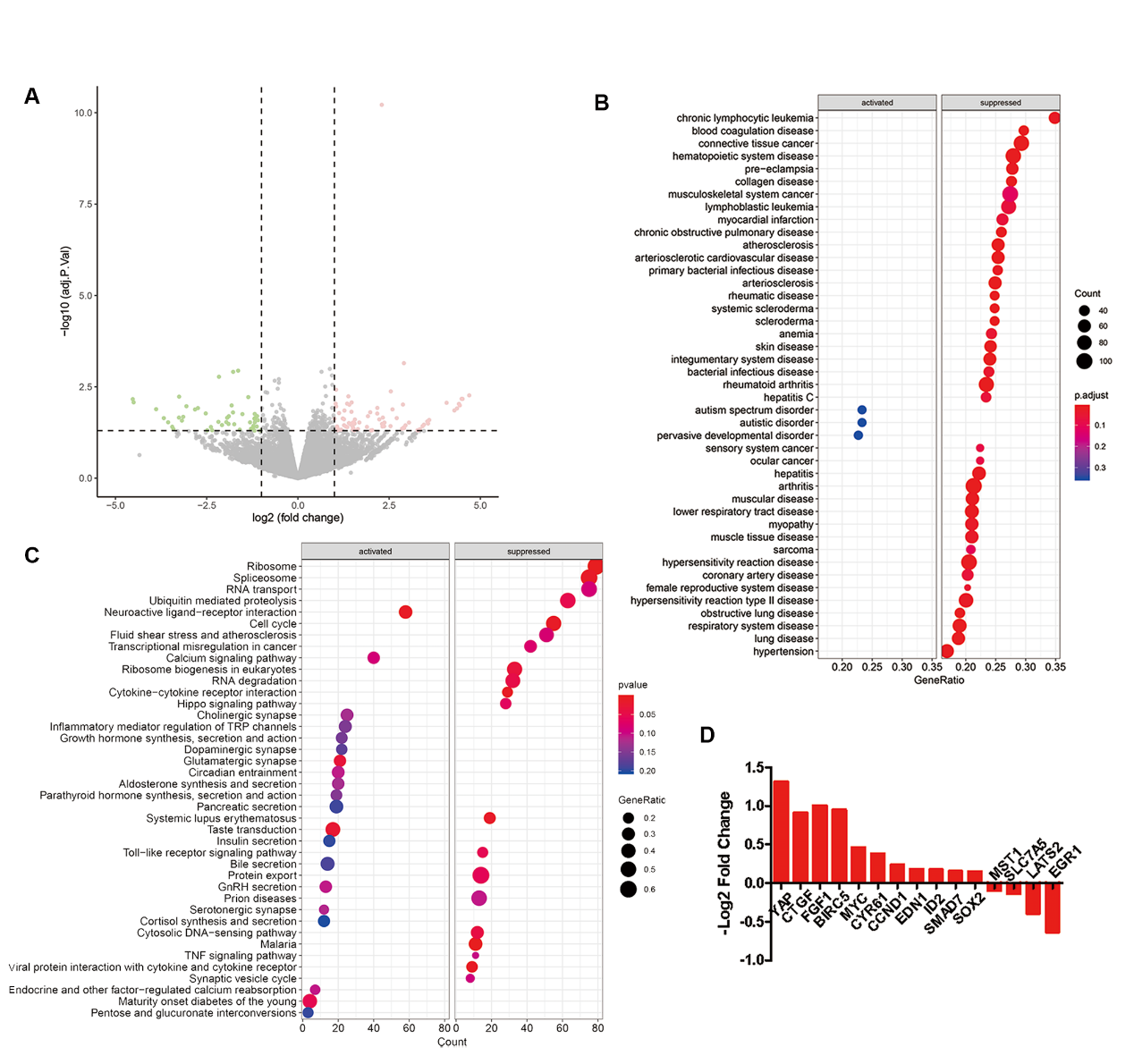


**Supplementary Figure 2.** Bioinformatics analysis of RNA-seq data.SK-N-AS cell were transfected with siPrp19 for 48h, and then subjected to RNA sequencing. **(A)** Volcano plot showed distribution of differential genes after absence of Prp19((|Fold of changes|>1.5, FDR<0.05). **(B)** Disease Ontology (DO) analysis enriched the affected diseases after Prp19 knockdown. **(C)** Kyoto Encyclopedia of Genes and Genomes (KEGG) enriched the affected pathways after Prp19 knockdown, and it indicated that the Hippo signaling pathway was suppressed. **(D)** RNA-seq analysed the mRNA level of YAP and its downstream genes after Prp19 knockdown. It indicated that YAP and almost its downstream genes decreased following Prp19 down regulation.


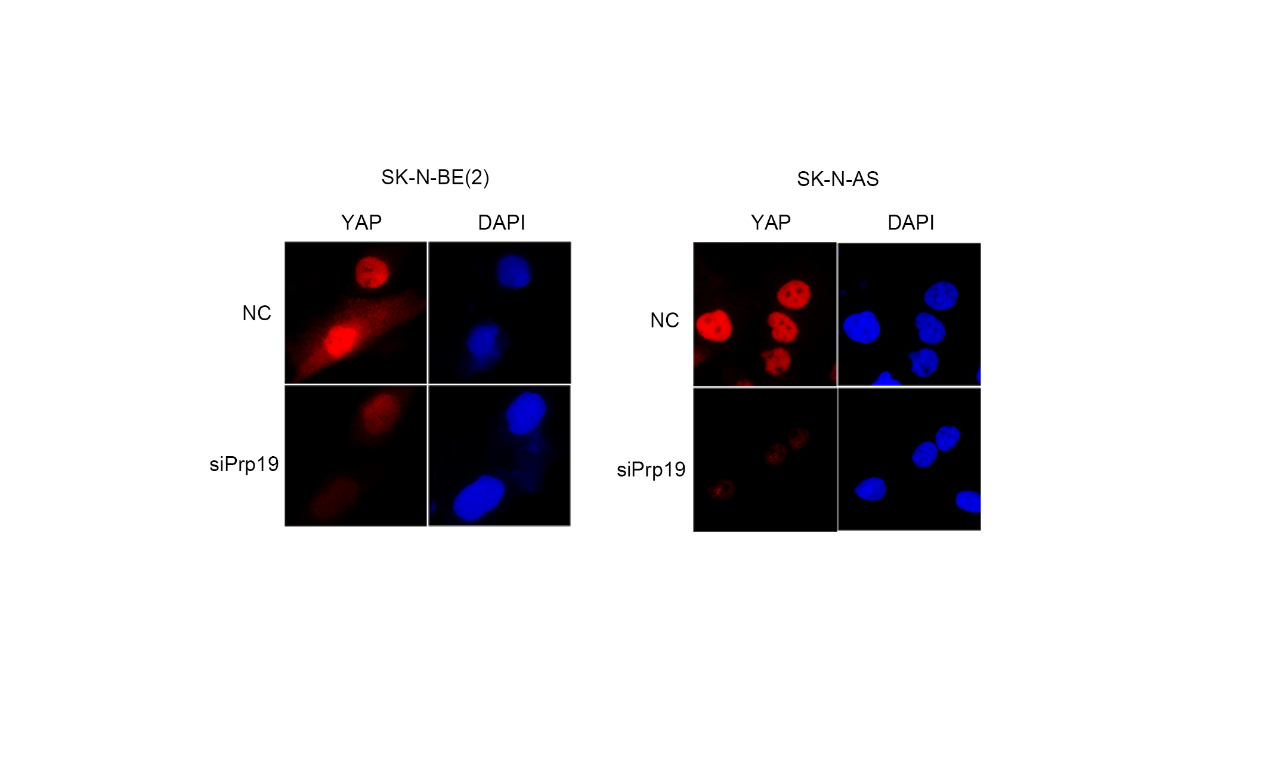


**Supplementary Figure 3.** Prp19 knockdown causes YAP decrease. SK-N-BE (2) and SK-N-AS cells were transfected with siPrp19 for 48h, then cells were immunostained with YAP antibody together with DAPI. Prp19 knockdown caused YAP decrease both in cytoplasm and nucleus.

- 1. **Supplementary Tables**

Supplemental Table 1 Correlation analysis between clinical characteristics and expression of Prp19 in the NRC dataset

| **Covariates** | **Total** | **Prp19** | | | ***χ²*** | | ***P*** |
| --- | --- | --- | --- | --- | --- | --- | --- |
|  |  | **high** | **low** | |  | |  |
| **Age at diagnosis** |  |  |  |  | |  | |
| ≥18months  ＜18months | 134  149 | 62  23 | 72  162 | 31.916 | | ＜0.001 | |
| **Clinical stages** |  |  |  |  | |  | |
| Ⅰ-Ⅱ Ⅳ-S | 113 | 16 | 97 |  | |  | |
| Ⅲ-Ⅳ | 167 | 69 | 98 | 23.512 | | ＜0.001 | |
| ***MYCN* state** |  |  |  |  | |  | |
| Amplification | 55 | 33 | 22 |  | |  | |
| Non-amplification | 222 | 50 | 172 | 29.502 | | ＜0.001 | |

Supplemental Table 2 Univariate analyses in the SEQC dataset

| Covariates | | OS EFS | | | | | | |
| --- | --- | --- | --- | --- | --- | --- | --- | --- |
|  | HR(95% CI) | | *P* | | | HR (95% CI) | *P* | |
| Prp19expression  (high vs low) | | 6.380(4.245-9.588) | | 0.0001 | 3.269(2.440-4.379) | | | ＜0.0001 |

Supplemental Table 3 Multivariable analyses in the SEQC dataset

| **Covariates** | | **OS EFS** | | | | | |
| --- | --- | --- | --- | --- | --- | --- | --- |
|  |  | HR (95% CI) | | *P* | HR (95% CI) | | *P* |
| Prp19 expression  (high vs low) | 2.395(1.545-3.712) | | ＜0.0001 | | 1.802(1.301-2.498) | ＜0.0001 | |
| Clinical stages  (Ⅰ-Ⅱ Ⅳ-S vs Ⅲ-Ⅳ) | 5.506(2.608-11.625) | | ＜0.0001 | | 2.726(1.816-4.091) | ＜0.0001 | |
| Age at diagnosis  (＜＞18months) | 3.057(1.805-5.177) | | ＜0.0001 | | 1.545(1.103-2.163) | | 0.011 |

Supplemental Table 4 Univariate analyses in the NRC dataset

| **Covariates** | | **OS EFS** | | | | | | | |
| --- | --- | --- | --- | --- | --- | --- | --- | --- | --- |
|  | | | HR (95% CI) | | *P* | | HR (95% CI) | | *P* |
| Prp19 expression  (high vs low) | 4.268(2.689-6.733) | | | ＜0.0001 | | 2.624(1.652-4.166) | | ＜0.0001 | |

Supplemental Table 5 Multivariable analyses in the NRC dataset

| **Covariates** | | | **OS EFS** | | | | | | | | |
| --- | --- | --- | --- | --- | --- | --- | --- | --- | --- | --- | --- |
|  |  |  | HR (95% CI) | | *P* | | | | HR (95% CI) | *P* | |
| Prp19 expression  (high vs low) | | 2.233(1.380-3.614) | | | 0.001 | | | 1.979(1.218-3.214) | | | 0.006 |
| Clinical stages  (Ⅰ-Ⅱ Ⅳ-S vs Ⅲ-Ⅳ) | 11.468(3.400-38.685) | | | ＜0.0001 | | 7.213(2.172-23.949) | | | | | 0.001 |
| Age at diagnosis  (＜＞18months) | 2.212(1.169-4.817) | | | | 0.015 | | 1.368(0.724-2.586) | | | 0.334 | |
